# The impact of composite AUC estimates on the prediction of systemic exposure in toxicology experiments

**DOI:** 10.1007/s10928-015-9413-5

**Published:** 2015-04-14

**Authors:** Tarjinder Sahota, Meindert Danhof, Oscar Della Pasqua

**Affiliations:** 1 Division of Pharmacology, Leiden Academic Centre for Drug Research, University of Leiden, Leiden, The Netherlands; 2 Clinical Pharmacology Modelling and Simulation, GlaxoSmithKline, Stockley Park West, Uxbridge, UK; 3 Clinical Pharmacology & Therapeutics, School of Life and Medical Sciences, University College London, London, UK

**Keywords:** Toxicokinetics, NOAEL, Safety margin, Model-based drug development

## Abstract

Current toxicity protocols relate measures of systemic exposure (i.e. AUC, C_max_) as obtained by non-compartmental analysis to observed toxicity. A complicating factor in this practice is the potential bias in the estimates defining safe drug exposure. Moreover, it prevents the assessment of variability. The objective of the current investigation was therefore (a) to demonstrate the feasibility of applying nonlinear mixed effects modelling for the evaluation of toxicokinetics and (b) to assess the bias and accuracy in summary measures of systemic exposure for each method. Here, simulation scenarios were evaluated, which mimic toxicology protocols in rodents. To ensure differences in pharmacokinetic properties are accounted for, hypothetical drugs with varying disposition properties were considered. Data analysis was performed using non-compartmental methods and nonlinear mixed effects modelling. Exposure levels were expressed as area under the concentration versus time curve (AUC), peak concentrations (C_max_) and time above a predefined threshold (TAT). Results were then compared with the reference values to assess the bias and precision of parameter estimates. Higher accuracy and precision were observed for model-based estimates (i.e. AUC, C_max_ and TAT), irrespective of group or treatment duration, as compared with non-compartmental analysis. Despite the focus of guidelines on establishing safety thresholds for the evaluation of new molecules in humans, current methods neglect uncertainty, lack of precision and bias in parameter estimates. The use of nonlinear mixed effects modelling for the analysis of toxicokinetics provides insight into variability and should be considered for predicting safe exposure in humans.

**Electronic supplementary material:**

The online version of this article (doi:10.1007/s10928-015-9413-5) contains supplementary material, which is available to authorized users.

## Introduction

**Fig. 1:**
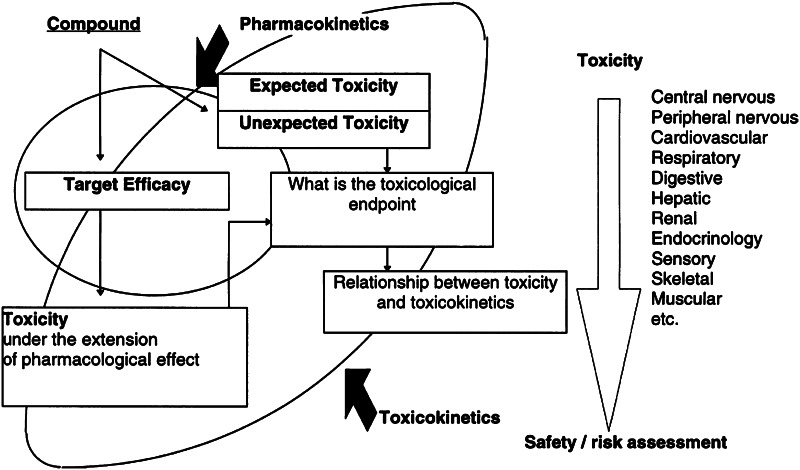
Diagram displaying the contribution of toxicokinetics and pharmacology for the characterisation of target-related adverse events and safety risk assessment. The circle depicting target efficacy highlights the role of information regarding the primary target engagement for safety risk assessment. Data on the target efficacy is usually obtained during in vitro and in vivo screening. The arrow indicates that inferences can be made about safety and risk based on the evidence from drug exposure and organ-specific toxicity data. Reprinted with permission from Horii 1998 [3]

The purpose of toxicokinetic studies in the evaluation of safety pharmacology and toxicity is the prediction of the risk that exposure to a new chemical or biological entity represents to humans [1, 2]. Understanding of the relationships between drug exposure, target engagement (i.e., activation or inhibition) and downstream biological effects of a given physiological pathway can provide insight into the mechanisms underlying both expected and ‘unexpected’ toxicity [3] (Fig. 1). In addition, the use of a mechanism-based approach has allowed better interpretation of time-dependencies in drug effect, which are often observed following chronic exposure to a drug (e.g., delayed toxicity) [4, 5].

**Fig. 2:**
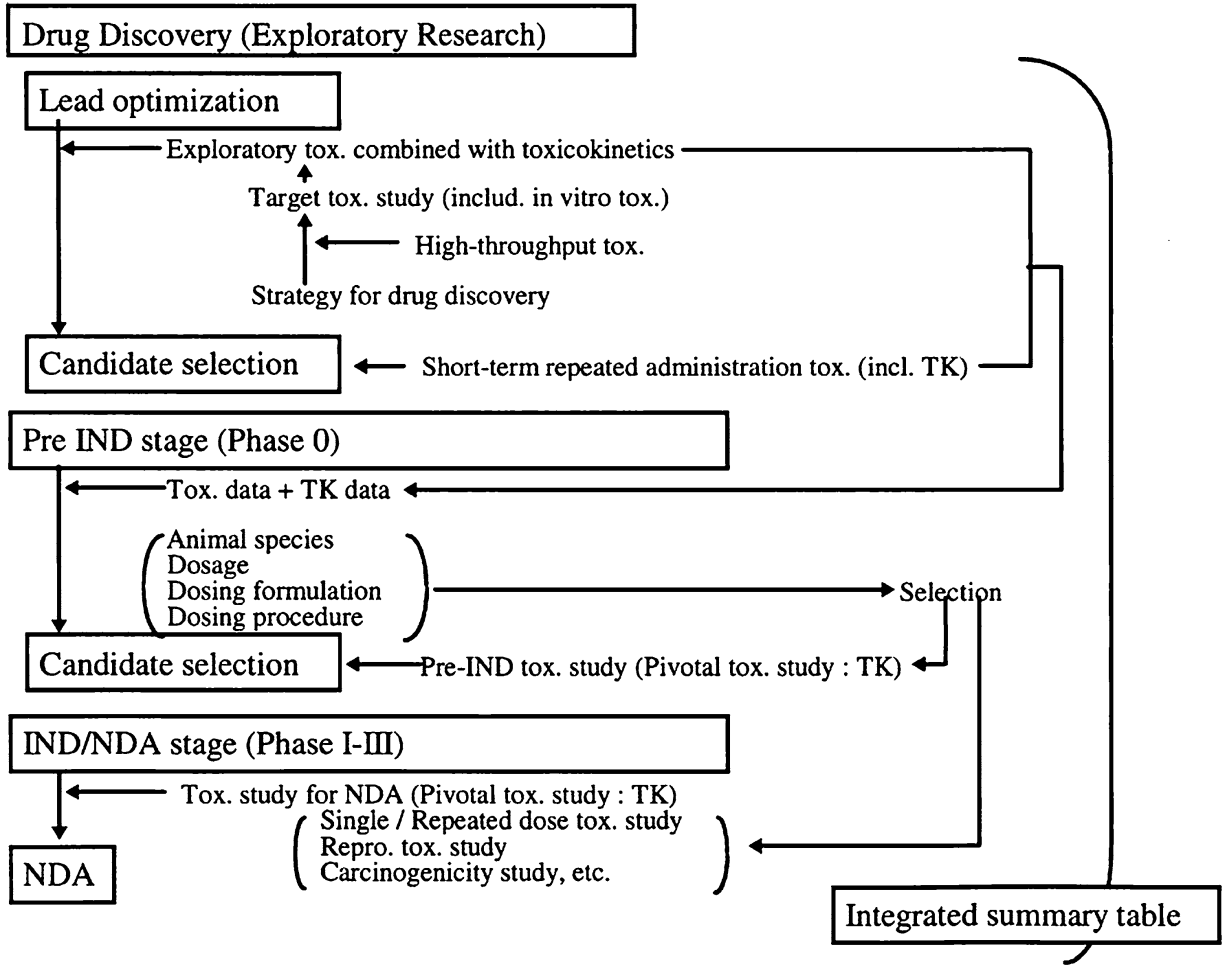
General toxicity data generated to support early clinical trials is gathered in the pre-IND/CTX stage. After IND/CTX submission, the regulatory agency will confirm whether adequate evidence of safety has been generated for human trials. Parameters derived from toxicokinetic data, such as the NOAEL, play a key role in the approval of protocols for first-time-in human studies. *IND/CTX* investigational new drug application, *NDA* new drug application, *TK* toxicokinetic study. Reprinted with permission from Horii 1998 [3]

Despite the increased attention to the importance of toxicokinetics in drug discovery and during the early stages of clinical development, the extrapolation and prediction of a safe exposure range in humans from preclinical experiments continues to be one of the major challenges in R&D (Fig. 2) [6]. Irrespective of the choice of experimental protocol, a common practice in toxicology remains the assessment of empirical safety thresholds, in particular the no observed adverse effect level (NOAEL), which is a *qualitative* indicator of acceptable risk. Even though support for the existence of thresholds has been argued on biological grounds [7–9], the NOAEL has been used to establish the safe exposure levels in humans. In fact, this threshold represents a proxy for another threshold, i.e., the underlying no adverse event level (NAEL).

The definition of the NOAEL varies from source to source [6]. Its calculation involves the determination of the lowest observed adverse effect level (LOAEL), which is the lowest observed dose level for which AEs are recorded. The NOAEL is the dose level below this. If no LOAEL is found, then the NOAEL cannot be determined. Usually, in the assessment of the LOAEL measures of systemic exposure are derived, such as area under the concentration versus time curve (AUC) and peak concentrations (C_max_), which serve as basis for the maximum allowed exposure in dose escalation studies in humans [10]. The aforementioned practices in safety and toxicity evaluation are driven by regulatory guidance [11, 12]. The scope of these guidances is to ensure that data on the systemic exposure achieved in animals is assessed in conjunction with dose level and its relationship to the time course of the toxicity or adverse events (Fig. 2). Another important objective is to establish the relevance of these findings for clinical safety as well as to provide information aimed at the optimisation of subsequent non-clinical toxicology studies.

Whilst the scope and intent of such guidance are well described since 1994, when it was introduced by the ICH, there has been much less attention to requirements for the analysis and interpretation of the data. In fact, precise details on the design of toxicokinetic studies or the statistical methods for calculating or estimating the endpoints or variables of interest, are not specified [13–15]. Instead, the assessment of exposure often takes places in satellite groups, which may not necessarily present the (same) adverse events or toxicity observed in the main experimental group. This is because of interferences associated with blood sampling procedures, which may affect toxicological findings. For this same reason, blood sampling for pharmacokinetics is often sparse [16]. Such practice also diverges from efforts in models in environmental toxicology, a field in which deterministic, physiologically-based pharmacokinetic models have been used for a long time [17, 18].

As a consequence, safety thresholds are primarily derived from inferences about the putative pharmacokinetic profiles in the actual treatment group. Furthermore, these thresholds rely on the accuracy of composite profiles obtained from limited sampling in individual animals. Composite profiles consist of pooled concentration data, which is averaged per time point under the assumption that inter-individual differences are simply residual variability, rather than intrinsic differences in pharmacokinetic processes [19]. Pharmacokinetic parameters such as area-under-concentration-time (AUC) and observed peak concentrations (C_max_) can then be either derived from the composite profile or by averaging individual estimates from serial profiles in satellite animals when frequent sampling schemes are feasible. Given that the parameters of interest are expressed as point estimates, within- and between-subject variability as well as uncertainty in estimation are not accounted for. In addition, pharmacokinetic data generated from different experiments are not evaluated in an integrated manner, whereby drug disposition (e.g., clearance) can be described mechanistically or at least compartmentally in terms of both first and zero order processes. This is further complicated by another major limitation in the way exposure is described by naïve pooling approaches, i.e., the impossibility to accurately derive parameters such as cumulative exposure, which may be physiologically a more relevant parameter for late onset or cumulative effects (e.g. lead toxicity, aminoglycosides) [20, 21]. Time spent above a threshold concentration may also bear greater physiological relevance for drugs which cause disruption of homeostatic feedback mechanisms. Such parameters cannot be described by empirical approaches due to limitations in sampling frequency.

By contrast, population pharmacokinetic-pharmacodynamic methodologies have the potential to overcome most of the aforementioned problems. Whilst the application of modelling in the evaluation of efficacy is widespread and well-established across different therapeutic areas [22–24], current practices have undoubtedly hampered the development of similar approaches for the evaluation of adverse events, safety pharmacology and toxicity. It should be noted that in addition to the integration of knowledge from a biological and pharmacological perspective, population models provide the basis for the characterisation of different sources of variability, allowing the identification of between-subject and between-occasion variability in parameters [25]. These random effects do not only reflect the evidence of statistical distributions. They can be used for inference about the mechanisms underlying adverse events and toxicity. In fact, recent advancements in environmental toxicology have shown the advantages of PBPK/PD modelling as a tool for quantifying target organ concentrations and dynamic response to arsenic in preclinical species [26].

The aim of this investigation was therefore to assess the relative performance of model-based approaches as compared to empirical methods currently used to analyse toxicokinetic data. We show that, modelling is an iterative process which allows further insight into relevant biological processes as well as into data gaps, providing the basis for experimental protocol optimisation. We illustrate the concepts by exploring a variety of scenarios in which hypothetical drugs with different disposition properties are evaluated.

## Methods

Using historical reference data from a range of non-steroidal anti-inflammatory compounds for which pharmacokinetic parameter estimates were known in rodents, a model-based approach was used to simulate the outcomes of a 3-month study protocol, in which toxicokinetic data for three hypothetical drugs were evaluated. The selection of non-steroidal anti-inflammatory compounds as paradigm for this analysis is due to the mechanisms underlying both short and long term adverse events as well as the evidence for a correlation between drug levels and incidence of such events in humans. In fact, a relationship has been identified between the degree of inhibition of cyclooxygenase at the maximum plasma concentration (C_max_) of individual non-steroidal anti-inflammatory drugs and relative risk (RR) of upper gastrointestinal bleeding/perforation [27].

### Simulation of drug profiles using predefined pharmacokinetic models

The impact of differences in drug disposition on bias and precision of the typical measures of systemic exposure was explored by including three different scenarios based on a one-compartment pharmacokinetics with linear and nonlinear (Michaelis–Menten) elimination as well as a two-compartment pharmacokinetics. Parameter values for each scenario are shown in Table 1. In all simulation scenarios, residual variability was set to 15 %. For the purposes of this exercise, we have assumed that the models used as reference show no misspecification. In addition, we have considered the use of a homogeneous population of rodents, avoiding the need to explore covariate relationships in any of the models.

**Table 1:** Pharmacokinetic models used to assess the impact of varying disposition properties on the estimation of safety thresholds

*Model A*: One-compartment model (1 CMT)		
Parameter	Pop estimate	BSV (%)
KA (h^−1^)	13.46	50
V (ml/kg)	49.4	16
CL (ml/h)	2.72	20

*Model B*: One compartment model with Michaelis–Menten elimination (1 CMT + MM). Parameter values were chosen to ensure departure from dose proportionality at the highest dose		
Parameter	Pop estimate	BSV (%)
V_max_ (mg/h)	2.72	20
Km (mg/ml)	1	–
Ka (h^−1^)	13.46	50
V (ml/kg)	49.4	16

*Model C*: Two-compartment model (2 CMT). The values for the absorption and elimination rate constants were selected in such a way that slow accumulation of drug is observed at stead-state conditions after daily dosing for approximately 2 weeks		
Parameter	Value	Variability (%)
Ka (h^−1^)	0.55	50
V (ml/kg)	49.4	16
CL (ml/h)	2.72	20
K12 (h^−1^)	0.3	–
K21 (h^−1^)	0.05	32

### Experimental design

**Fig. 3:**
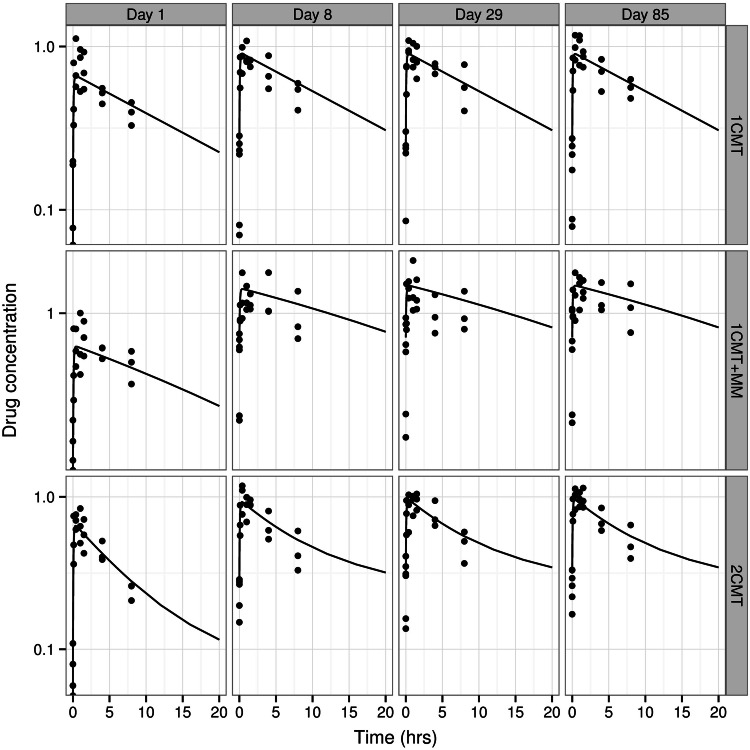
Overview of a simulated dataset along with the predicted pharmacokinetic profiles for each of the experimental scenarios, in which blood samples are collected from 3 animals per sampling time point. *Dots* represent simulated concentrations at the pre-defined sampling times, whereas the *solid black line* depicts the population predicted profile after a dose of 30 mg/kg for hypothetical drugs with different pharmacokinetic characteristics

Experimental procedures were defined according to current guidelines for the assessment of toxicity. A summary of the sampling schemes and experimental conditions is shown in Table 2. The protocol design for each experiment with the three hypothetical drugs was based on protocols typically used for chronic toxicity evaluation. Four treatment groups receiving oral daily doses of vehicle, 10, 30, and 100 mg/kg/day were tested throughout this set of virtual experiments. The same treatment groups were present in all duration cohorts (1 week, 1 month or 3 months). Satellite groups each were used to characterise the pharmacokinetics under the dosing conditions in the animals used for the assessment of toxicity. This procedure ensures the availability of more frequent blood samples for toxicokinetics, while not influencing the assessment of the toxicity. Two different sampling schedules were investigated, namely, composite sampling and serial sampling. For the sake of comparison, the same number of samples was collected in both cases. For composite sampling, blood was collected from three animals in the satellite group at predetermined sampling time points, namely, 0.1, 0.4, 1, 1.5, 4, 8, 24 h after drug administration on sampling days (see Table 2). The allocation of animals to each sampling time point was random within the constraint that all animals were sampled an equal number of times. An overview of a simulated dataset along with the predicted pharmacokinetic profiles for each of the experimental scenarios is shown in Fig. 3.

**Table 2:** Experimental design characteristics of treatment and satellite groups in a general toxicity study with serial and composite pharmacokinetic sampling

Duration	Numbers of animals	Sampling scheme
1 week	*Toxicity*: 4 per dose group *Satellite*: 3 per dose group	*Toxicity*: Composite 2 per animal *Satellite*: Serial profiles from day 1 only
1 month	*Toxicity*: 10 per dose group *Satellite*: 3 per dose group	*Toxicity*: Composite 2 per animal *Satellite*: Serial profiles from day 1 and 28
3 months	*Toxicity*: 12 per dose group *Satellite*: 3 per dose group	*Toxicity*: Composite: week 4, week 13 *Satellite*: Serial profiles from day 1, week 4, week 13

### Derivation of true exposure levels

Five different measures of exposure were derived from the simulated concentration profiles obtained from the models used for simulation. They included the 24-h area under the concentration versus time curve (AUC), the maximum concentration (C_max_), the time above a threshold drug concentration (TAT), the predicted 6-month cumulative AUC and the predicted 6-month C_max_. These exposure measures can be seen alongside the formula used for their calculation in Table 3. The threshold for adverse events was assumed to be 10 μg/ml. This arbitrary value was selected for illustrative purposes only. The simulations (*n* = 200 replicates) were performed assuming repeat dosing for up to 6 months (3 months beyond the treatment duration presented the investigated studies) in order to evaluate the implications of longer periods of drug exposure.

**Table 3:** Exposure measures derived from the simulated concentration vs. time profiles. The assessment of bias and precision in the estimates of safety thresholds was based on these secondary pharmacokinetic parameters, which are shown alongside the formula used for their calculation. Individual predicted drug concentrations are denoted by *C*_*p*_(*t*)

Variable name	Symbol	Model based exposure calculation
24-h AUC	AUC24	$\mathop \smallint \limits_{t - 24}^{t} C_{p} dt$
24-h C_max_	C_max24_	${ \hbox{max} }\left( {\left\{ {C_{p} \left( s \right):t - 24 < s < t} \right\}} \right)$
24-h time above threshold drug concentration	TAT	$\mathop \smallint \limits_{t - 24}^{t} 1_{{C_{p} > thresh}} dt$
Predicted 6-month cumulative AUC	CAUC	$\mathop \smallint \limits_{0}^{6 months} C_{p} dt$
Predicted 6-month C_max_	C_max24_	${ \hbox{max} }\left( {\left\{ {C_{p} \left( s \right):0 < s < 6 months} \right\}} \right)$

### Calculation of measures of exposure by non-compartmental analysis

Data from composite sampling across all satellite animals were used to determine the overall drug exposure, which consisted in averaging the simulated concentrations at each sampling time point. A similar approach was used for serial sampling, but in this case, drug exposure was calculated for each individual animal and then averaged over the cohort. In both cases, the arithmetic mean and geometric mean were used as summary statistics. As non-compartmental methods do not allow extrapolation beyond the actual experimental conditions, only three of the five measures of exposure were derived, namely, the AUC, estimated using the linear-logarithmic trapezoidal rule, the C_max_, and the TAT.

### Calculation of measures of exposure by nonlinear mixed effects modelling

For each simulation replicate, drug concentration profiles were fitted to pharmacokinetic models using the first-order conditional estimation method with interaction (FOCEI), as implemented in NONMEM. Model building steps were limited to the same structural models used for the initial simulations under the assumption that pharmacokinetic properties of the drugs are known at the time toxicology experiments are performed. Model convergence was determined by successful minimisation and estimation of the covariance step. Data below the lower quantification limit (BQL) were omitted to mimic experimental conditions in which imputation methods are not applied. Estimates for all five measures of exposure were calculated by using same procedures applied for the reference values obtained during the initial simulation step (see Table 3).

### Comparison

To ensure accurate estimates of bias and precision of the two methodologies, the process of simulation and estimation of exposure (using non-compartmental vs. nonlinear mixed effects) was repeated 200 times. Bias and precision were assessed by the relative error, scaled relative mean error (SRME) and the coefficient of variation (CV) respectively [28]:

$$ SMRE = \frac{1}{N}\mathop \sum \limits_{i = 1}^{N} \frac{{\left( {estimated_{i} - true} \right)}}{true} \times 100 $$

$$ CV = \frac{1}{N}\sqrt {\mathop \sum \limits_{i = 1}^{N} \left( {\frac{{estimated_{i} - mean}}{mean}} \right)^{2} } \times 100 $$

All simulations and fitting procedures described above were performed in NONMEM 7.1 [29]. Data manipulation and statistical and graphical summaries were performed in R 3.0.0 [30].

## Results

The use of simulated data for the evaluation of hypothetical scenarios provided clear insight of the impact of current practices on the accuracy and precision of safety thresholds, and in particular of the NOAEL. Irrespective of the use of serial or sparse sampling schemes for the characterisation of the concentration versus time profiles, model convergence rates were usually high, with successful completion of the covariance step. An overview of the convergence rates is presented in Table 4.

**Table 4:** Rates of convergence and covariance (parameter precision) estimation based on nonlinear mixed effects modelling. Simulated drug concentrations collected at the predefined sampling times were used as input for the pharmacokinetic analysis

Model	Successful convergence	Successful covariance step
1 CMT	99.75	99.75
1 CMT + MM	99.75	99.75
2 CMT	100	100

To facilitate the comparison of the magnitude of bias and precision, results from modelling are shown together with the parameter values obtained from non-compartmental analysis where applicable. Due to the large number of experimental conditions to be summarised, here we present a brief description of the relative errors obtained in the 3-month protocol, for AUC, C_max_ and TAT. All other experimental conditions, including an overview of the scaled relative mean error (SRME) and the coefficient of variation (CV) are presented in tabular format as supplemental material (Table S1).

**Fig. 4:**
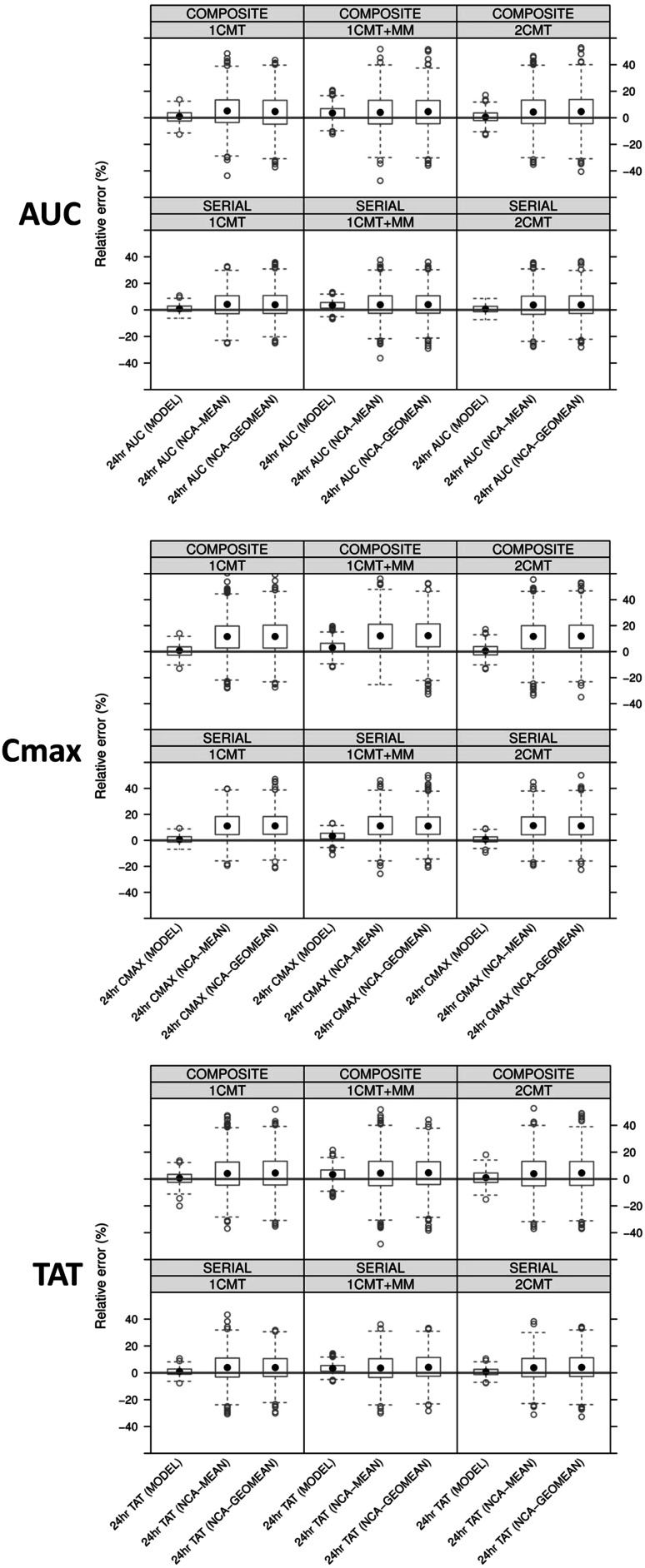
Relative errors of parameter estimates for AUC (*upper panel*), C_max_ (*mid panel*) and TAT (*lower panel*). Data refers only to the 3-month toxicology protocol design following administration of 30 mg/kg/day of three hypothetical drugs with different pharmacokinetic profiles. Similar results were found for other cohorts in which 10 and 100 mg/kg/day were evaluated. *Dots* represent the median, *boxes* show the 25th and 75th percentiles, *error bars* denote the 5th and 95th percentiles. The *red line* shows the reference level for relative error equal to zero. *Composite* composite sampling, *GEOMEAN* geometric mean, *MEAN* arithmetic mean, *MODEL* nonlinear mixed effects modelling, *NCA* non-compartmental analysis and *Serial* serial sampling (Color figure online)

**Fig. 5:**
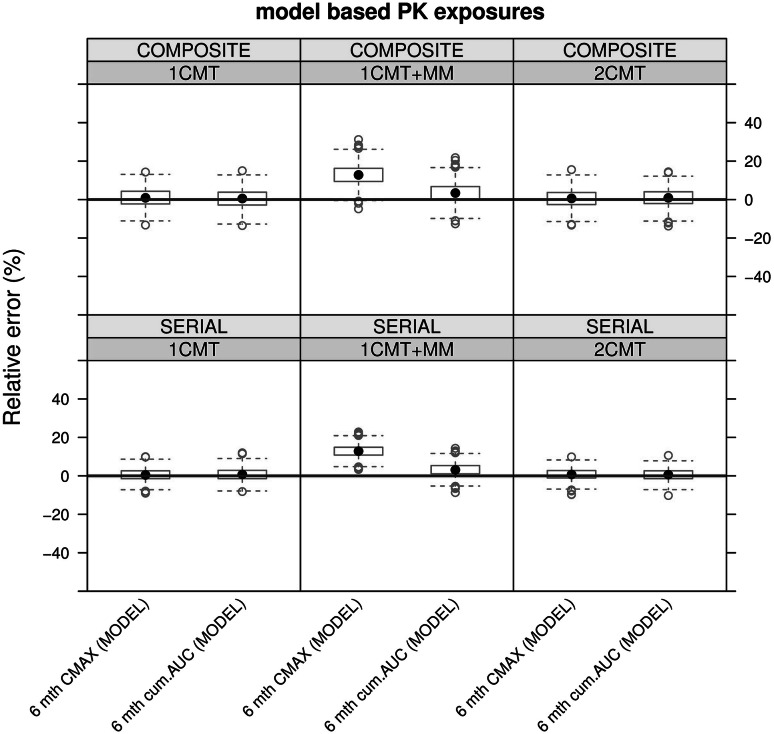
Overview of the relative errors of model-based estimators of long-term exposure, i.e. predicted peak concentrations after 6 months (6 mth Cmax) and cumulative area under the concentration vs. time curve (6 mth cum. AUC). The analysis is based on the data from a 3-month toxicology protocol following administration of 30 mg/kg/day of three hypothetical drugs with different pharmacokinetic profiles. Similar results were found for other cohorts in which 10 and 100 mg/kg/day were evaluated. *Dots* represent the median, *boxes* show the 25th and 75th percentiles, *error bars* denote 1.5 times the interquartile range from the median. The *red line* shows the reference level for relative error equal to zero (Color figure online)

In Fig. 4, the relative errors are presented for the estimates for AUC, C_max_ and TAT. The relative errors were clearly smaller when measures of exposure were derived by modelling, as compared to the results obtained by non-compartmental analysis. In fact, the accuracy and precision of model-based estimates for all three measures of exposure were similar across the different dosing groups and treatment durations. Non-compartmental estimates of exposure showed significantly higher bias and less precision in all scenarios. The performance for model-based exposure estimates derived from the 3-month protocol is summarised in Fig. 5.

Our results also reveal the impact of composite versus serial sampling on bias and precision. For both model-based and non-compartmental methods, the coefficient of variation increased with composite designs (with 8 animals), as compared to serial sampling designs (with 3 animals). However, the increase in precision for non-compartmental method was larger than for model-based estimates. It should also be noted that C_max_ was consistently over-estimated by the non-compartmental method. We also demonstrate that the use of arithmetic and geometric means for NCA had minor impact in these relatively small groups.

Lastly, it was found that that nonlinearity in pharmacokinetics also has an important effect on bias and precision when sparse samples and limited number of dose levels are evaluated experimentally. Model-based estimates in the 1 CMT + MM scenario showed increased bias compared to the 1 CMT and 2 CMT scenarios.

## Discussion

In this investigation we have attempted to identify important limitations in the use of non-compartmental methods for the analysis of toxicokinetic data. Irrespective of the limited number of scenarios, our findings illustrate the feasibility of using hierarchical models for the evaluation of toxicokinetic data using a well-established parameterisation for drug disposition processes. Furthermore, given that model performance in the analysis of toxicokinetic data has been previously evaluated [31], we have been able to focus on the performance of measures of exposure that cannot be derived from empirical approaches, i.e., non-compartmental methods [32].

It is important to highlight that the use of compartmental models, instead of physiologically-based pharmacokinetic models in this exercise was required to avoid issues such as parameter identifiability [33], which would arise from the data generated in standard toxicology protocols. The plasma pharmacokinetic profiles derived for the hypothetical compounds were considered realistic enough to reflect the time course of drug levels observed in many toxicology studies. In fact, these profiles are greatly affected by the standard sampling schemes in toxicology experiments, which may not allow one to identify more than one- and two-compartment models. Moreover, consideration was given to the implications that high doses may have on drug metabolism and elimination. A pharmacokinetic model with Michaelis–Menten elimination was also included to ensure accurate characterisation of dose- and concentration-dependent pharmacokinetics, which is likely to occur for many compounds at least in one experimental dose level. Saturation of metabolism has implications for the interpretation of safety thresholds, especially if nonlinearity is not observed at pharmacologically relevant levels. The results presented here should therefore be indicative of the most common toxicokinetic profiles. Given the evidence of the superiority of nonlinear mixed effects modelling to describe sparse pharmacokinetic data [34–37], we anticipate the possibility to generalise the lessons learned to a much wider range of drugs, for which pharmacokinetic parameter values may differ considerably from those presented here.

### Parameter precision and bias

As shown in Table 4, the high convergence rates of models and high success rate for the computation of the covariance matrix for the scenarios tested here confirm the robustness of results obtained using nonlinear mixed-effects modelling. Despite variations in bias and precision, parameter precision was consistently high. Whilst these results must be interpreted under the assumption of minor or no model misspecification, the use of modelling showed particularly good performance (CV < 10 % and SRME < 10 % for within study exposure predictions and SRME < 15 % for long term exposure predictions). Such high levels of precision may not be required for safe exposure evaluation where between-subject variability in humans is expected to be larger and comparatively large uncertainty factors are routinely used. This suggests that a model-based approach will enable considerable reductions in the numbers of animals and/or samples to be used in experimental protocols whilst providing acceptable parameter precision. Moreover since optimal design methodologies for model-based analysis are well established, further refinement of the experimental protocol design is feasible if experimentalists and statisticians choose nonlinear mixed effects modelling as the primary method of analysis.

On the other hand, the presence of bias in some of the experimental conditions presented here has clear implications for the so-called safety margin and toxicological cover to be used as proxy for risk during clinical development, especially for C_max_, which is consistently over-estimated. This is due to the definition of peak concentrations in non-compartmental analysis where C_M_ is necessarily greater than or equal to $Cp\left( {t = {\text{T}}_{ \hbox{max} } } \right)$, where T_max_ represents the time point which maximises the true concentration–time profile. When the sampling scheme contains other observations in the region of T_max_ there is potential for neighbouring sampling times to produce higher than predicted concentrations due to natural variability. This is a fundamental limitation in the methodology in that more samples around T_max_ which intuitively should increase confidence, actually leads to more bias. In other words, with non-compartmental analysis precisely estimating T_max_ comes at the unavoidable cost of biased estimation of C_max_. Model-based analysis has an additional advantage in this respect. Without model misspecification issues, maximum likelihood estimates are (asymptotically) unbiased and have the property that increased sampling uniformly increases precision. The implications of model specification issues are discussed further in the limitations section. Given that the residual variability in the scenarios was not large (i.e., fixed at 15 %), the bias seen here may increase with larger residual noise, which may occur in real life. The issue of bias can be further mitigated by the appropriate use of predictive checks. An example of the procedures can be found in the supplemental material for naproxen [38], where predictive checks including data analysed by non-compartmental methods illustrate how to assess bias in AUC and C_max_.

### Data integration

In contrast to non-compartmental methods, our investigation was based on an integrated analysis of the data, i.e. by combining the results from all experimental cohorts. This is undoubtedly the primary driver of the increased accuracy and precision in model-based estimates [38–40]. In fact, we envisage further improvement by incorporating pharmacokinetic data from other experiments in the same species, which are normally collected during the preclinical evaluation of a molecule, as for instance during the characterisation of drug metabolism. Such an increase in precision would represent further adherence to the reduction, refinement and replacement principle (3 Rs) in ethical animal studies [41, 42]. It should also be noted that the possibility of data integration provides the basis for combining safety pharmacology and adverse event data, enabling the development of toxicokinetic-toxicodynamic models and consequently allowing for the evaluation of exposure–response relationships in a continuous manner. Such models would represent an advancement in toxicology and risk management and mitigation, as they provide the basis for mechanism-based inferences about unwanted effects, irrespective of their incidence or occurrence in the actual experimental protocol [4, 43].

It is important to realise that the typical point estimates of parameters derived from empirical methods to describe drug exposure give an undue measure of certainty, allowing for the propagation of uncertainty from estimation to uncertainty in safety thresholds, such as NOAEL. Whilst there exist methods for estimating uncertainty in a composite or destructive sampling approach [44–46], their adoption in experimental research has not been widespread due in part to the requirement of normality assumptions on toxicokinetic parameters, and an acceptance in guidelines towards possibly large amounts of imprecision [12].

As demonstrated here, model-based methods allow simulations to be performed in conjunction with estimation procedures, enabling the assessment of uncertainty associated with a variety of causes such as uninformative study design, large variability and/or unknown covariates. This entails an increase in the quality of the decision-making process and ultimately in the interpretation of the estimated safety thresholds [47].

Given the success of modelling and simulation in drug development [48–50], one should ask why the field of toxicology has yet to embrace it. The scepticism regarding the value of model-based approaches often arises from a view that knowledge about the model is required in advance [51, 52]. This argumentation is however flawed. Non-linear mixed effects modelling is specifically intended to efficiently process sparse data. The performance of the model-based exposure estimates in the composite designs is illustrative of this. Moreover, the inference principles used for hypothesis generation and characterisation of drug disposition parameters relies on the use of statistical criteria that are sophisticated enough to allow model identification and its suitability for subsequent parameter estimation purposes. Moreover, it should be noted that non-compartmental methods also make implicit assumptions about the underlying concentration versus time profile. For instance, with a linear-logarithmic analysis of AUC, first-order elimination kinetics is assumed. The suitability of measures of central tendency will also depend on the assumed distribution characteristics and on residual variability. These assumptions are often implicit and their validity regarding the dataset at hand cannot be checked during the analysis. There are no strong statistical justification to support the choice for non-compartmental methods, other than the lack of technical knowledge and familiarity with hierarchical modelling by toxicologists in industry and regulatory agencies. The persistence in the use of non-compartmental methods bears an unnoticed cost, i.e., the ethical cost of utilising more animals than what is really necessary.

### Potential limitations

In the present investigation, the impact of model misspecification in the analysis of general toxicity data was not investigated. For exposure measures which have a corresponding estimate based on non-compartmental methods (e.g. AUC and C_max_), the impact is likely to be small as long as the model fit to the data is good. This is because these measures are highly dependent on the observations. Therefore, accurate prediction of the observed profiles during model evaluation is likely to result in accurate prediction of these exposure variables. Model misspecification however, may lead to significant bias when exposure predictions are made outside the experimental context (i.e. longer timescales or different dosing regimens) [53, 54]. This is a risk when the pharmacokinetics of the drug is nonlinear or shows metabolic saturation. To mitigate such effects we recommend that model selection criteria take into account not only the ability to describe data, but also the physiological relevance of model assumptions. When model development ends in multiple competing models performing similarly with respect to statistical selection criteria, clear reporting of such model uncertainty is necessary. Model averaging should be discouraged when predictions arising from different model differ significantly [55]. Finally, parameter uncertainty should be incorporated into the predictions of exposure to ensure accurate evaluation of risk and potential therapeutic window of the compound.

In summary, evaluation of safety is paramount for the progression of new molecules into humans. Historically, toxicology experiments have evolved based the assumption that experimental findings suffice to demonstrate the absence or presence of risk. This assumption disregards growing evidence of the advantages of data integration for the characterisation of drug properties. Whilst the challenges R&D faces to translate toxicity findings from animals to humans may remain, the use of an integrated approach to the analysis and interpretation of toxicokinetic data represents further adherence to the 3Rs principle, enabling significant reduction in number of animals required for the evaluation of toxicokinetics.

## Electronic supplementary material

Below is the link to the electronic supplementary material.

Supplementary material 1 (DOCX 373 kb)

Supplementary material 2 (PDF 97 kb)

## Abbreviations

AUC: Area under the concentration versus time curve
C_max_: Peak concentrations
ICH: International conference on harmonisation of technical requirements for registration of pharmaceuticals for human use
PD: Pharmacodynamics
PK: Pharmacokinetics
TAT: Time above a concentration threshold
