# Supplementary material for: The impact of composite AUC estimates on the prediction of systemic exposure in toxicology experiments
Source: J Pharmacokinet Pharmacodyn. 2015 Apr 14;42(3):251–61. doi: 10.1007/s10928-015-9413-5 (PMC4432106; doi:10.1007/s10928-015-9413-5)
Supplement: Supplementary file 1 — Supplementary material 1 (DOCX 373 kb) [file 10928_2015_9413_MOESM1_ESM.docx]

**Supplemental material**


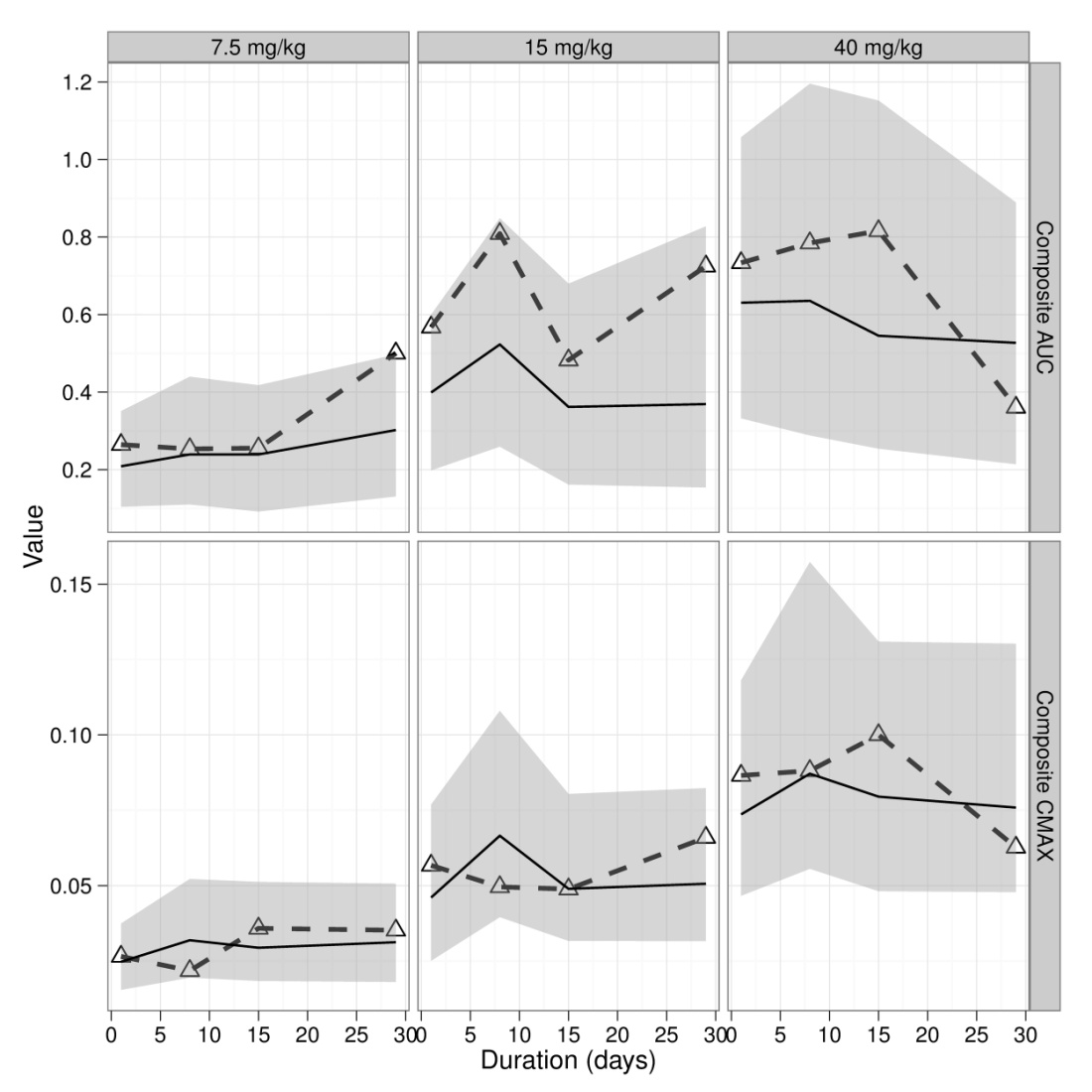


**Figure 1S: Example of a predictive check for estimating bias in secondary measures of drug exposure (AUC and C_MAX_).** The model presented here has similar parameter values and is structurally related to the hypothetical compounds described in the manuscript. Experimental protocol and data analysis details can be found in Sahota et al. (38). Observed exposures derived from non-compartmental methods (triangles and dotted line) are overlaid with 95% prediction distribution (shaded region). The predictive checks reveal no signficant model misspecification in terms of predicted observed AUCs and C_MAX_ vaues***.***

**
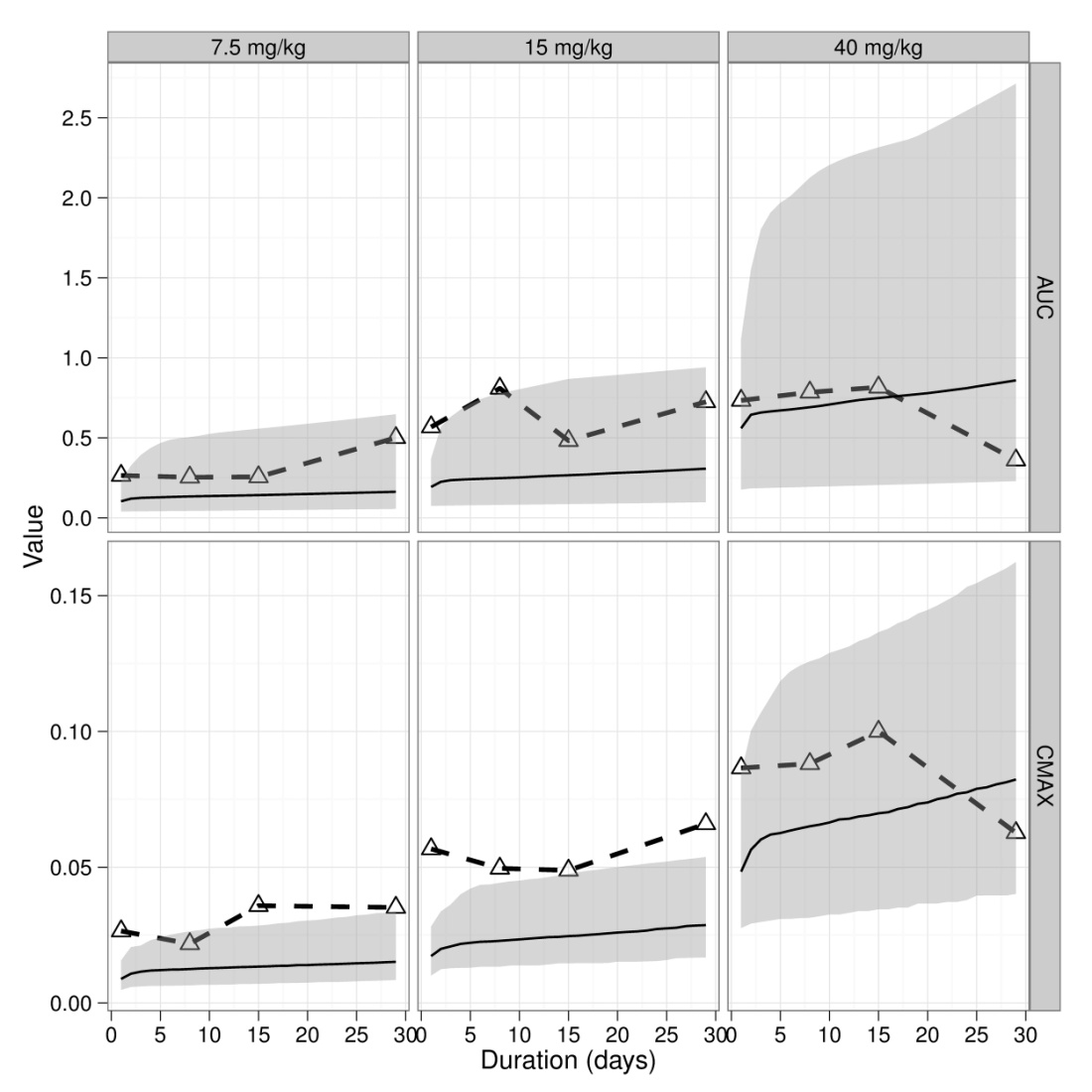
**

**Figure 2S: Model-predicted vs. non-compartmental estimates of AUC and C_MAX_.** Observed exposures (triangles and dotted line) obtained by non-compartmental analysis are overlaid with model predicted estimates throughout the duration of the treatment. The median and 95% prediction interval are shown as solid line and shaded regions in each panel. . Experimental protocol and data analysis details can be found in Sahota et al. (38). These results highlight the discrepancies between C_MAX_ values obtained by pharmacokinetic modeling (solid line) and derived by non-compartmental analysis (dotted line). Since model-based C_MAX_ values are not significantly biased (see Figure 1S), such a difference implies considerable bias in the estimates of C_MAX_ from non-compartmental analysis.
