# Supplementary material for: The impact of composite AUC estimates on the prediction of systemic exposure in toxicology experiments
Source: J Pharmacokinet Pharmacodyn. 2015 Apr 14;42(3):251–61. doi: 10.1007/s10928-015-9413-5 (PMC4432106; doi:10.1007/s10928-015-9413-5)
Supplement: Supplementary file 2 — Supplementary material 2 (PDF 97 kb) [file 10928_2015_9413_MOESM2_ESM.pdf]

**Supplemental Material**

**Table S1 -** Overview of SRME %and CV% values for all simulation scenarios, cohorts and analysis methods. SAMP – sampling scheme (serial vs. sparse); LABEL – indicates the secondary pharmacokinetic measure of exposure; DOSE - daily dose in mg/kg; WEEK – duration of the treatment; SRME% - scaled relative mean error in percentage; CV% - coefficient of variation in percentage; NCA- non-compartmental analysis; MEAN – arithmetic mean; GEOMEAN- geometric mean.

| MODEL | SAMP | LABEL | DOSE | WEEK | SRME% : MODEL | CV% : MODEL | SRME% : NCA-MEAN | CV% : NCA-MEAN | SRME% : NCA-GEOMEAN | CV% : NCA-GEOMEAN |
|---|---|---|---|---|---|---|---|---|---|---|
| 1CMT | SERIA | 24hr AUC | 10 | 1 | 0.8066 | 2.787 | 3.993 | 10.74 | 4.15 | 9.824 |
| 1CMT | SERIA | 24hr AUC | 10 | 4 | 1.403 | 2.982 | 5.107 | 9.256 | 4.053 | 10.26 |
| 1CMT | SERIA | 24hr AUC | 10 | 12 | 0.784 | 2.822 | 3.055 | 9.367 | 3.948 | 9.826 |
| 1CMT | SERIA | 24hr AUC | 30 | 1 | 0.8066 | 2.787 | 4.565 | 9.472 | 4.298 | 9.344 |
| 1CMT | SERIA | 24hr AUC | 30 | 4 | 1.403 | 2.982 | 3.721 | 9.74 | 4.934 | 10.58 |
| 1CMT | SERIA | 24hr AUC | 30 | 12 | 0.784 | 2.822 | 3.065 | 9.873 | 4.258 | 10.48 |
| 1CMT | SERIA | 24hr AUC | 100 | 1 | 0.8066 | 2.787 | 4.615 | 9.845 | 3.538 | 9.214 |
| 1CMT | SERIA | 24hr AUC | 100 | 4 | 1.403 | 2.982 | 3.02 | 9.474 | 3.939 | 8.846 |
| 1CMT | SERIA | 24hr AUC | 100 | 12 | 0.784 | 2.822 | 4.038 | 9.928 | 3.59 | 10.71 |
| 1CMT | COM | 24hr AUC | 10 | 1 | 0.4114 | 4.651 | 5.245 | 13.41 | 4.815 | 12.93 |
| 1CMT | COM | 24hr AUC | 10 | 4 | 0.6365 | 4.324 | 5.634 | 12.73 | 3.838 | 13.51 |
| 1CMT | COM | 24hr AUC | 10 | 12 | 1.115 | 4.373 | 3.815 | 13.17 | 4.564 | 12.98 |
| 1CMT | COM | 24hr AUC | 30 | 1 | 0.4114 | 4.651 | 6.231 | 12.56 | 4.027 | 13.28 |
| 1CMT | COM | 24hr AUC | 30 | 4 | 0.6365 | 4.324 | 5.212 | 12.68 | 4.35 | 12.98 |
| 1CMT | COM | 24hr AUC | 30 | 12 | 1.115 | 4.373 | 3.564 | 13.24 | 4.632 | 12.19 |
| 1CMT | COM | 24hr AUC | 100 | 1 | 0.4114 | 4.651 | 5.29 | 12.85 | 4.466 | 13.68 |
| 1CMT | COM | 24hr AUC | 100 | 4 | 0.6365 | 4.324 | 4.209 | 12.18 | 3.833 | 13.03 |
| 1CMT | COM | 24hr AUC | 100 | 12 | 1.115 | 4.373 | 4.564 | 12.71 | 3.903 | 13.63 |

| MODEL | SAMP | LABEL | DOSE | WEEK | SRME% : MODEL | CV% : MODEL | SRME% : NCA-MEAN | CV% : NCA-MEAN | SRME% : NCA-GEOMEAN | CV% : NCA -GEOMEAN |
|---|---|---|---|---|---|---|---|---|---|---|
| 1CMT+MM | SERIAL | 24hr AUC | 10 | 1 | 3.454 | 3.152 | 4.042 | 9.245 | 4.066 | 9.429 |
| 1CMT+MM | SERIAL | 24hr AUC | 10 | 4 | 3.177 | 3.255 | 3.784 | 9.352 | 5.347 | 8.897 |
| 1CMT+MM | SERIAL | 24hr AUC | 10 | 12 | 3.457 | 3.167 | 4.354 | 9.667 | 3.889 | 9.966 |
| 1CMT+MM | SERIAL | 24hr AUC | 30 | 1 | 3.165 | 3.309 | 3.883 | 10.35 | 3.277 | 9.953 |
| 1CMT+MM | SERIAL | 24hr AUC | 30 | 4 | 3.416 | 3.281 | 4.477 | 10.15 | 3.199 | 10.62 |
| 1CMT+MM | SERIAL | 24hr AUC | 30 | 12 | 3.572 | 3.226 | 3.282 | 11.27 | 4.358 | 9.756 |
| 1CMT+MM | SERIAL | 24hr AUC | 100 | 1 | 2.993 | 3.069 | 4.03 | 9.883 | 3.608 | 10.66 |
| 1CMT+MM | SERIAL | 24hr AUC | 100 | 4 | 3.369 | 3.37 | 4.945 | 9.871 | 3.363 | 9.435 |
| 1CMT+MM | SERIAL | 24hr AUC | 100 | 12 | 3.459 | 3.024 | 3.657 | 9.409 | 5.191 | 9.725 |
| 1CMT+MM | COMPO | 24hr AUC | 10 | 1 | 3.176 | 4.932 | 3.502 | 12.07 | 5.057 | 13.09 |
| 1CMT+MM | COMPO | 24hr AUC | 10 | 4 | 3.192 | 4.768 | 3.777 | 12.38 | 4.02 | 12.54 |
| 1CMT+MM | COMPO | 24hr AUC | 10 | 12 | 3.399 | 4.88 | 3.864 | 13.15 | 4.442 | 12.16 |
| 1CMT+MM | COMPO | 24hr AUC | 30 | 1 | 4.214 | 5.316 | 3.68 | 13.83 | 3.438 | 12.73 |
| 1CMT+MM | COMPO | 24hr AUC | 30 | 4 | 3.262 | 5.196 | 5.853 | 12.8 | 4.427 | 12.42 |
| 1CMT+MM | COMPO | 24hr AUC | 30 | 12 | 3.496 | 5.407 | 3.888 | 13.18 | 7.559 | 13.46 |
| 1CMT+MM | COMPO | 24hr AUC | 100 | 1 | 3.868 | 5.214 | 4.33 | 12.11 | 3.738 | 13.41 |
| 1CMT+MM | COMPO | 24hr AUC | 100 | 4 | 3.502 | 4.82 | 5.149 | 13.12 | 2.725 | 13.61 |
| 1CMT+MM | COMPO | 24hr AUC | 100 | 12 | 3.312 | 4.733 | 3.812 | 12.58 | 5.262 | 12.38 |

| MODEL | SAMP | LABEL | DOSE | WEEK | SRME% : MODEL | CV% : MODEL | SRME% : NCA-MEAN | CV% : NCA-MEAN | SRME% : NCA-GEOMEAN | CV% : NCA-GEOMEAN |
|---|---|---|---|---|---|---|---|---|---|---|
| 2CMT | SERIAL | 24hr AUC | 10 | 1 | 0.5484 | 2.819 | 3.009 | 10.21 | 4.508 | 9.854 |
| 2CMT | SERIAL | 24hr AUC | 10 | 4 | 0.8587 | 3.256 | 4.84 | 10.01 | 5.346 | 9.577 |
| 2CMT | SERIAL | 24hr AUC | 10 | 12 | 0.5528 | 2.943 | 3.429 | 9.583 | 3.767 | 10.48 |
| 2CMT | SERIAL | 24hr AUC | 30 | 1 | 0.5484 | 2.819 | 4.379 | 10.31 | 3.721 | 9.561 |
| 2CMT | SERIAL | 24hr AUC | 30 | 4 | 0.8587 | 3.256 | 3.079 | 10.8 | 3.865 | 9.479 |
| 2CMT | SERIAL | 24hr AUC | 30 | 12 | 0.5528 | 2.943 | 3.966 | 10.32 | 4.311 | 9.826 |
| 2CMT | SERIAL | 24hr AUC | 100 | 1 | 0.5484 | 2.819 | 4.732 | 10.38 | 3.649 | 10.28 |
| 2CMT | SERIAL | 24hr AUC | 100 | 4 | 0.8587 | 3.256 | 2.261 | 9.635 | 3.11 | 9.859 |
| 2CMT | SERIAL | 24hr AUC | 100 | 12 | 0.5528 | 2.943 | 4.047 | 10.43 | 3.432 | 9.983 |
| 2CMT | COMPO | 24hr AUC | 10 | 1 | 0.6988 | 4.286 | 2.96 | 13.31 | 4.885 | 13.99 |
| 2CMT | COMPO | 24hr AUC | 10 | 4 | 0.8458 | 4.847 | 5.062 | 12.59 | 6.413 | 12.44 |
| 2CMT | COMPO | 24hr AUC | 10 | 12 | 0.5273 | 4.514 | 5.177 | 12.45 | 4.647 | 13.75 |
| 2CMT | COMPO | 24hr AUC | 30 | 1 | 0.6988 | 4.286 | 4.258 | 13.9 | 4.463 | 12.16 |
| 2CMT | COMPO | 24hr AUC | 30 | 4 | 0.8458 | 4.847 | 3.757 | 12.9 | 2.889 | 14.08 |
| 2CMT | COMPO | 24hr AUC | 30 | 12 | 0.5273 | 4.514 | 4.381 | 12.81 | 4.018 | 12.59 |
| 2CMT | COMPO | 24hr AUC | 100 | 1 | 0.6988 | 4.286 | 5.057 | 13.23 | 3.703 | 13.07 |
| 2CMT | COMPO | 24hr AUC | 100 | 4 | 0.8458 | 4.847 | 3.908 | 14.25 | 6.301 | 13.07 |
| 2CMT | COMPO | 24hr AUC | 100 | 12 | 0.5273 | 4.514 | 4.586 | 12.94 | 4.2 | 12.94 |

| MODEL | SAMP | LABEL | DOSE | WEEK | SRME% : MODEL | CV% : MODEL | SRME% : NCA-MEAN | CV% : NCA-MEAN | SRME% : NCA-GEOMEAN | CV% : NCA-GEOMEAN |
|---|---|---|---|---|---|---|---|---|---|---|
| 1CMT | SERIAL | 24hr CMAX | 10 | 1 | 0.7306 | 2.899 | 11.54 | 9.945 | 10.8 | 9.747 |
| 1CMT | SERIAL | 24hr CMAX | 10 | 4 | 0.5798 | 2.893 | 10.74 | 9.382 | 11.41 | 10.37 |
| 1CMT | SERIAL | 24hr CMAX | 10 | 12 | 0.8339 | 3.045 | 11.58 | 10.51 | 10.32 | 9.832 |
| 1CMT | SERIAL | 24hr CMAX | 30 | 1 | 0.7306 | 2.899 | 11.72 | 8.873 | 11.42 | 9.831 |
| 1CMT | SERIAL | 24hr CMAX | 30 | 4 | 0.5798 | 2.893 | 10.6 | 9.846 | 12.38 | 10.74 |
| 1CMT | SERIAL | 24hr CMAX | 30 | 12 | 0.8339 | 3.045 | 9.94 | 10.25 | 11.82 | 10.21 |
| 1CMT | SERIAL | 24hr CMAX | 100 | 1 | 0.7306 | 2.899 | 11.92 | 8.828 | 11.23 | 10.8 |
| 1CMT | SERIAL | 24hr CMAX | 100 | 4 | 0.5798 | 2.893 | 11.55 | 10.67 | 12.12 | 9.98 |
| 1CMT | SERIAL | 24hr CMAX | 100 | 12 | 0.8339 | 3.045 | 12.1 | 10.27 | 11.61 | 9.536 |
| 1CMT | COMPO | 24hr CMAX | 10 | 1 | 0.6322 | 4.557 | 10.61 | 12.68 | 11.85 | 12.11 |
| 1CMT | COMPO | 24hr CMAX | 10 | 4 | 0.647 | 4.659 | 9.818 | 12.3 | 11.59 | 14.16 |
| 1CMT | COMPO | 24hr CMAX | 10 | 12 | 0.3693 | 4.73 | 9.869 | 13.41 | 10.37 | 13.76 |
| 1CMT | COMPO | 24hr CMAX | 30 | 1 | 0.6322 | 4.557 | 12.7 | 12.29 | 10.56 | 13.33 |
| 1CMT | COMPO | 24hr CMAX | 30 | 4 | 0.647 | 4.659 | 12.34 | 12.77 | 11.76 | 13.51 |
| 1CMT | COMPO | 24hr CMAX | 30 | 12 | 0.3693 | 4.73 | 12.21 | 13.67 | 13.15 | 13.48 |
| 1CMT | COMPO | 24hr CMAX | 100 | 1 | 0.6322 | 4.557 | 12.12 | 12 | 10.8 | 12.03 |
| 1CMT | COMPO | 24hr CMAX | 100 | 4 | 0.647 | 4.659 | 11.19 | 13.89 | 12.08 | 12.21 |
| 1CMT | COMPO | 24hr CMAX | 100 | 12 | 0.3693 | 4.73 | 9.968 | 13.04 | 11.67 | 12.97 |

| MODEL | SAMP | LABEL | DOSE | WEEK | SRME% : MODEL | CV% : MODEL | SRME% : NCA-MEAN | CV% : NCA-MEAN | SRME% : NCA-GEOMEAN | CV% : NCA-GEOMEAN |
|---|---|---|---|---|---|---|---|---|---|---|
| 1CMT+MM | SERIA | 24hr CMAX | 10 | 1 | 2.57 | 3.176 | 16.96 | 8.955 | 6.995 | 8.341 |
| 1CMT+MM | SERIA | 24hr CMAX | 10 | 4 | 3.916 | 3.461 | 7.387 | 13.15 | 14.32 | 15.12 |
| 1CMT+MM | SERIA | 24hr CMAX | 10 | 12 | 1.814 | 2.586 | 15.26 | 6.701 | 11.92 | 8.455 |
| 1CMT+MM | SERIA | 24hr CMAX | 30 | 1 | 2.997 | 2.533 | 12.89 | 9.863 | 6.667 | 7.948 |
| 1CMT+MM | SERIA | 24hr CMAX | 30 | 4 | 2.195 | 3.013 | 11.01 | 12.71 | 13.32 | 8.925 |
| 1CMT+MM | SERIA | 24hr CMAX | 30 | 12 | 2.997 | 4.675 | 12.79 | 7.512 | 11.2 | 9.27 |
| 1CMT+MM | SERIA | 24hr CMAX | 100 | 1 | 2.539 | 2.99 | 9.542 | 6.405 | 4.643 | 10.42 |
| 1CMT+MM | SERIA | 24hr CMAX | 100 | 4 | 2.236 | 4.535 | 13.72 | 9.105 | 13.44 | 11.59 |
| 1CMT+MM | SERIA | 24hr CMAX | 100 | 12 | 3.108 | 3.104 | 8.219 | 10.94 | 16.4 | 9.72 |
| 1CMT+MM | COM | 24hr CMAX | 10 | 1 | 4.193 | 3.414 | 22.85 | 17.18 | 12.08 | 13.98 |
| 1CMT+MM | COM | 24hr CMAX | 10 | 4 | 3.428 | 6.017 | 13.7 | 14.06 | 13.61 | 14.29 |
| 1CMT+MM | COM | 24hr CMAX | 10 | 12 | 3.076 | 4.608 | 14.43 | 17.99 | 13.83 | 15.88 |
| 1CMT+MM | COM | 24hr CMAX | 30 | 1 | 3.89 | 4.507 | 16.05 | 12.46 | 17.38 | 18.27 |
| 1CMT+MM | COM | 24hr CMAX | 30 | 4 | 2.88 | 3.301 | 17.05 | 12 | 17.62 | 10.72 |
| 1CMT+MM | COM | 24hr CMAX | 30 | 12 | 4.101 | 7.039 | 12.73 | 17.45 | 14.67 | 10.93 |
| 1CMT+MM | COM | 24hr CMAX | 100 | 1 | 5.108 | 3.725 | 15.38 | 10.59 | 14.11 | 16.02 |
| 1CMT+MM | COM | 24hr CMAX | 100 | 4 | 0.8694 | 3.595 | 10.94 | 9.899 | 10.14 | 8.477 |
| 1CMT+MM | COM | 24hr CMAX | 100 | 12 | 2.504 | 6.026 | 8.776 | 13.82 | 13.18 | 17.37 |

| MODEL | SAMP | LABEL | DOSE | WEEK | SRME% : MODEL | CV% : MODEL | SRME% : NCA-MEAN | CV% : NCA-MEAN | SRME% : NCA-GEOMEAN | CV% : NCA-GEOMEAN |
|---|---|---|---|---|---|---|---|---|---|---|
| 2CMT | SERIAL | 24hr CMAX | 10 | 1 | 0.7472 | 3.015 | 10.9 | 10.33 | 12.4 | 9.994 |
| 2CMT | SERIAL | 24hr CMAX | 10 | 4 | 0.8701 | 2.938 | 11.06 | 10.1 | 11.89 | 9.653 |
| 2CMT | SERIAL | 24hr CMAX | 10 | 12 | 0.6139 | 2.959 | 11.32 | 10.4 | 10.68 | 10.7 |
| 2CMT | SERIAL | 24hr CMAX | 30 | 1 | 0.7472 | 3.015 | 11.35 | 9.376 | 10.76 | 10.76 |
| 2CMT | SERIAL | 24hr CMAX | 30 | 4 | 0.8701 | 2.938 | 11.19 | 10.82 | 10.64 | 9.939 |
| 2CMT | SERIAL | 24hr CMAX | 30 | 12 | 0.6139 | 2.959 | 11.19 | 9.61 | 11.63 | 9.976 |
| 2CMT | SERIAL | 24hr CMAX | 100 | 1 | 0.7472 | 3.015 | 10.86 | 8.862 | 11.59 | 9.721 |
| 2CMT | SERIAL | 24hr CMAX | 100 | 4 | 0.8701 | 2.938 | 11.34 | 9.274 | 10.92 | 9.915 |
| 2CMT | SERIAL | 24hr CMAX | 100 | 12 | 0.6139 | 2.959 | 10.53 | 10.36 | 10.95 | 10.01 |
| 2CMT | COMPO | 24hr CMAX | 10 | 1 | 0.9441 | 4.693 | 10.16 | 14.9 | 9.881 | 12.51 |
| 2CMT | COMPO | 24hr CMAX | 10 | 4 | 0.7777 | 4.718 | 10.49 | 12.52 | 11.22 | 12.73 |
| 2CMT | COMPO | 24hr CMAX | 10 | 12 | 0.6393 | 5.002 | 11.36 | 14.06 | 11.31 | 13.68 |
| 2CMT | COMPO | 24hr CMAX | 30 | 1 | 0.9441 | 4.693 | 11.82 | 14.28 | 13.13 | 12.84 |
| 2CMT | COMPO | 24hr CMAX | 30 | 4 | 0.7777 | 4.718 | 12.67 | 12.09 | 12.69 | 12.89 |
| 2CMT | COMPO | 24hr CMAX | 30 | 12 | 0.6393 | 5.002 | 11.43 | 13.28 | 11.23 | 12.88 |
| 2CMT | COMPO | 24hr CMAX | 100 | 1 | 0.9441 | 4.693 | 11.76 | 11.98 | 11.6 | 14.2 |
| 2CMT | COMPO | 24hr CMAX | 100 | 4 | 0.7777 | 4.718 | 12.52 | 13.27 | 12.34 | 11.92 |
| 2CMT | COMPO | 24hr CMAX | 100 | 12 | 0.6393 | 5.002 | 10.43 | 13.29 | 12.31 | 13.51 |

| MODEL | SAMP | LABEL | DOSE | WEEK | SRME% : MODEL | CV% : MODEL | SRME% : NCA-MEAN | CV% : NCA-MEAN | SRME% : NCA-GEOMEAN | CV% : NCA-GEOMEAN |
|---|---|---|---|---|---|---|---|---|---|---|
| 1CMT | SERIAL | 24hr TAT | 10 | 1 | 1.104 | 2.855 | 3.516 | 10.72 | 3.06 | 9.718 |
| 1CMT | SERIAL | 24hr TAT | 10 | 4 | 0.9731 | 2.95 | 3.725 | 9.746 | 4.782 | 9.684 |
| 1CMT | SERIAL | 24hr TAT | 10 | 12 | 0.7183 | 2.738 | 3.945 | 9.307 | 3.261 | 10.24 |
| 1CMT | SERIAL | 24hr TAT | 30 | 1 | 1.104 | 2.855 | 3.846 | 10.61 | 3.263 | 9.204 |
| 1CMT | SERIAL | 24hr TAT | 30 | 4 | 0.9731 | 2.95 | 3.112 | 10.22 | 4.793 | 10.36 |
| 1CMT | SERIAL | 24hr TAT | 30 | 12 | 0.7183 | 2.738 | 3.944 | 10.07 | 4.197 | 9.843 |
| 1CMT | SERIAL | 24hr TAT | 100 | 1 | 1.104 | 2.855 | 5.182 | 10.92 | 4.328 | 9.798 |
| 1CMT | SERIAL | 24hr TAT | 100 | 4 | 0.9731 | 2.95 | 4.906 | 10.05 | 3.802 | 10.18 |
| 1CMT | SERIAL | 24hr TAT | 100 | 12 | 0.7183 | 2.738 | 3.106 | 10.58 | 4.077 | 9.474 |
| 1CMT | COMPO | 24hr TAT | 10 | 1 | 0.1905 | 4.511 | 3.011 | 12.53 | 3.635 | 12.95 |
| 1CMT | COMPO | 24hr TAT | 10 | 4 | 1.022 | 4.251 | 4.875 | 12.75 | 5.447 | 12.87 |
| 1CMT | COMPO | 24hr TAT | 10 | 12 | 0.1963 | 4.784 | 6.422 | 12.15 | 4.639 | 12.27 |
| 1CMT | COMPO | 24hr TAT | 30 | 1 | 0.1905 | 4.511 | 2.807 | 13.83 | 4.701 | 13.07 |
| 1CMT | COMPO | 24hr TAT | 30 | 4 | 1.022 | 4.251 | 6.049 | 12.4 | 2.792 | 13.49 |
| 1CMT | COMPO | 24hr TAT | 30 | 12 | 0.1963 | 4.784 | 3.696 | 11.97 | 4.605 | 14.31 |
| 1CMT | COMPO | 24hr TAT | 100 | 1 | 0.1905 | 4.511 | 3.725 | 11.97 | 3.86 | 12.92 |
| 1CMT | COMPO | 24hr TAT | 100 | 4 | 1.022 | 4.251 | 4.092 | 13.38 | 5.199 | 13.95 |
| 1CMT | COMPO | 24hr TAT | 100 | 12 | 0.1963 | 4.784 | 2.898 | 13.19 | 3.727 | 12.68 |

| MODEL | SAMP | LABEL | DOSE | WEEK | SRME% : MODEL | CV% : MODEL | SRME% : NCA-MEAN | CV% : NCA-MEAN | SRME% : NCA-GEOMEAN | CV% : NCA-GEOMEAN |
|---|---|---|---|---|---|---|---|---|---|---|
| 1CMT+MM | SERIAL | 24hr TAT | 10 | 1 | 3.51 | 2.891 | 3.178 | 10.52 | 5.906 | 9.741 |
| 1CMT+MM | SERIAL | 24hr TAT | 10 | 4 | 3.492 | 3.447 | 4.244 | 10.57 | 4.249 | 10.57 |
| 1CMT+MM | SERIAL | 24hr TAT | 10 | 12 | 2.956 | 3.273 | 3.632 | 10.48 | 4.573 | 10.21 |
| 1CMT+MM | SERIAL | 24hr TAT | 30 | 1 | 3.473 | 3.036 | 2.326 | 10.07 | 4.185 | 9.986 |
| 1CMT+MM | SERIAL | 24hr TAT | 30 | 4 | 3.747 | 3.168 | 4.568 | 10.9 | 3.938 | 10.14 |
| 1CMT+MM | SERIAL | 24hr TAT | 30 | 12 | 2.577 | 3.079 | 3.717 | 9.856 | 2.89 | 10.71 |
| 1CMT+MM | SERIAL | 24hr TAT | 100 | 1 | 2.873 | 3.179 | 3.713 | 10.02 | 4.367 | 9.592 |
| 1CMT+MM | SERIAL | 24hr TAT | 100 | 4 | 3.447 | 2.947 | 3.826 | 10.31 | 4.132 | 9.726 |
| 1CMT+MM | SERIAL | 24hr TAT | 100 | 12 | 3.263 | 3.185 | 3.388 | 10.08 | 3.888 | 10.4 |
| 1CMT+MM | COMPO | 24hr TAT | 10 | 1 | 3.786 | 4.678 | 4.308 | 13.54 | 2.909 | 11.91 |
| 1CMT+MM | COMPO | 24hr TAT | 10 | 4 | 3.544 | 4.288 | 5.699 | 13.18 | 4.995 | 12.28 |
| 1CMT+MM | COMPO | 24hr TAT | 10 | 12 | 3.001 | 4.644 | 4.643 | 13.52 | 3.367 | 11.96 |
| 1CMT+MM | COMPO | 24hr TAT | 30 | 1 | 3.472 | 4.949 | 2.865 | 14.24 | 5.8 | 12.81 |
| 1CMT+MM | COMPO | 24hr TAT | 30 | 4 | 3.562 | 4.826 | 4.148 | 13.6 | 2.608 | 12.83 |
| 1CMT+MM | COMPO | 24hr TAT | 30 | 12 | 3.763 | 5.2 | 5.31 | 13.39 | 4.779 | 13.14 |
| 1CMT+MM | COMPO | 24hr TAT | 100 | 1 | 3.168 | 4.877 | 3.489 | 14.78 | 5.073 | 11.72 |
| 1CMT+MM | COMPO | 24hr TAT | 100 | 4 | 3.176 | 5.163 | 3.82 | 13.59 | 5.091 | 13.09 |
| 1CMT+MM | COMPO | 24hr TAT | 100 | 12 | 3.286 | 5.171 | 3.485 | 13.09 | 4.549 | 12.53 |

| MODEL | SAMP | LABEL | DOSE | WEEK | SRME% : MODEL | CV% : MODEL | SRME% : NCA-MEAN | CV% : NCA-MEAN | SRME% : NCA-GEOMEAN | CV% : NCA-GEOMEAN |
|---|---|---|---|---|---|---|---|---|---|---|
| 2CMT | SERIAL | 24hr TAT | 10 | 1 | 0.7214 | 3.072 | 5.377 | 10.74 | 4.592 | 10.37 |
| 2CMT | SERIAL | 24hr TAT | 10 | 4 | 0.5198 | 2.962 | 4.408 | 10.03 | 4.979 | 9.128 |
| 2CMT | SERIAL | 24hr TAT | 10 | 12 | 0.7113 | 2.8 | 2.965 | 10.73 | 5.301 | 10.43 |
| 2CMT | SERIAL | 24hr TAT | 30 | 1 | 0.7214 | 3.072 | 3.161 | 9.763 | 3.817 | 9.643 |
| 2CMT | SERIAL | 24hr TAT | 30 | 4 | 0.5198 | 2.962 | 3.984 | 9.413 | 4.117 | 9.773 |
| 2CMT | SERIAL | 24hr TAT | 30 | 12 | 0.7113 | 2.8 | 3.268 | 9.713 | 4.137 | 9.669 |
| 2CMT | SERIAL | 24hr TAT | 100 | 1 | 0.7214 | 3.072 | 3.36 | 10.37 | 4.292 | 10.07 |
| 2CMT | SERIAL | 24hr TAT | 100 | 4 | 0.5198 | 2.962 | 5.182 | 9.81 | 2.393 | 10.03 |
| 2CMT | SERIAL | 24hr TAT | 100 | 12 | 0.7113 | 2.8 | 3.436 | 10.14 | 3.129 | 9.555 |
| 2CMT | COMPO | 24hr TAT | 10 | 1 | 0.63 | 4.583 | 4.218 | 11.94 | 3.532 | 13.95 |
| 2CMT | COMPO | 24hr TAT | 10 | 4 | 0.9004 | 4.939 | 4.626 | 14.21 | 3.189 | 12.9 |
| 2CMT | COMPO | 24hr TAT | 10 | 12 | 1.214 | 4.747 | 3.387 | 14.45 | 4.046 | 12.71 |
| 2CMT | COMPO | 24hr TAT | 30 | 1 | 0.63 | 4.583 | 3.724 | 12.65 | 4.101 | 12.58 |
| 2CMT | COMPO | 24hr TAT | 30 | 4 | 0.9004 | 4.939 | 5.745 | 12.5 | 5.615 | 13.23 |
| 2CMT | COMPO | 24hr TAT | 30 | 12 | 1.214 | 4.747 | 4.28 | 12.28 | 4.449 | 13.19 |
| 2CMT | COMPO | 24hr TAT | 100 | 1 | 0.63 | 4.583 | 3.513 | 12.72 | 5.004 | 11.98 |
| 2CMT | COMPO | 24hr TAT | 100 | 4 | 0.9004 | 4.939 | 4.212 | 11.98 | 4.553 | 12.29 |
| 2CMT | COMPO | 24hr TAT | 100 | 12 | 1.214 | 4.747 | 2.435 | 13.04 | 5.184 | 14.3 |

| MODEL | SAMP | LABEL | DOSE | WEEK | SRME% : MODEL | CV% : MODEL |
|---|---|---|---|---|---|---|
| 1CMT | SERIAL | 6 mth CMAX | 10 | 1 | 0.3527 | 2.86 |
| 1CMT | SERIAL | 6 mth CMAX | 10 | 4 | 0.4266 | 3.159 |
| 1CMT | SERIAL | 6 mth CMAX | 10 | 12 | 0.5896 | 3.19 |
| 1CMT | SERIAL | 6 mth CMAX | 30 | 1 | 0.3527 | 2.86 |
| 1CMT | SERIAL | 6 mth CMAX | 30 | 4 | 0.4266 | 3.159 |
| 1CMT | SERIAL | 6 mth CMAX | 30 | 12 | 0.5896 | 3.19 |
| 1CMT | SERIAL | 6 mth CMAX | 100 | 1 | 0.3527 | 2.86 |
| 1CMT | SERIAL | 6 mth CMAX | 100 | 4 | 0.4266 | 3.159 |
| 1CMT | SERIAL | 6 mth CMAX | 100 | 12 | 0.5896 | 3.19 |
| 1CMT | SERIAL | 6 mth cum.AUC | 10 | 1 | 0.6984 | 2.862 |
| 1CMT | SERIAL | 6 mth cum.AUC | 10 | 4 | 0.7434 | 3.223 |
| 1CMT | SERIAL | 6 mth cum.AUC | 10 | 12 | 0.8577 | 3.317 |
| 1CMT | SERIAL | 6 mth cum.AUC | 30 | 1 | 0.6984 | 2.862 |
| 1CMT | SERIAL | 6 mth cum.AUC | 30 | 4 | 0.7434 | 3.223 |
| 1CMT | SERIAL | 6 mth cum.AUC | 30 | 12 | 0.8577 | 3.317 |
| 1CMT | SERIAL | 6 mth cum.AUC | 100 | 1 | 0.6984 | 2.862 |
| 1CMT | SERIAL | 6 mth cum.AUC | 100 | 4 | 0.7434 | 3.223 |
| 1CMT | SERIAL | 6 mth cum.AUC | 100 | 12 | 0.8577 | 3.317 |
| 1CMT | COMPOSITE | 6 mth CMAX | 10 | 1 | 1.031 | 4.775 |
| 1CMT | COMPOSITE | 6 mth CMAX | 10 | 4 | 0.8082 | 4.768 |
| 1CMT | COMPOSITE | 6 mth CMAX | 10 | 12 | 0.7882 | 4.773 |
| 1CMT | COMPOSITE | 6 mth CMAX | 30 | 1 | 1.031 | 4.775 |
| 1CMT | COMPOSITE | 6 mth CMAX | 30 | 4 | 0.8082 | 4.768 |
| 1CMT | COMPOSITE | 6 mth CMAX | 30 | 12 | 0.7882 | 4.773 |
| 1CMT | COMPOSITE | 6 mth CMAX | 100 | 1 | 1.031 | 4.775 |
| 1CMT | COMPOSITE | 6 mth CMAX | 100 | 4 | 0.8082 | 4.768 |
| 1CMT | COMPOSITE | 6 mth CMAX | 100 | 12 | 0.7882 | 4.773 |

| | | | | | | |
|---|---|---|---|---|---|---|
| 1CMT | COMPOSITE | 6 mth cum.AUC | 10 | 1 | 0.1272 | 4.794 |
| 1CMT | COMPOSITE | 6 mth cum.AUC | 10 | 4 | 0.808 | 4.862 |
| 1CMT | COMPOSITE | 6 mth cum.AUC | 10 | 12 | 0.7279 | 4.937 |
| 1CMT | COMPOSITE | 6 mth cum.AUC | 30 | 1 | 0.1272 | 4.794 |
| 1CMT | COMPOSITE | 6 mth cum.AUC | 30 | 4 | 0.808 | 4.862 |
| 1CMT | COMPOSITE | 6 mth cum.AUC | 30 | 12 | 0.7279 | 4.937 |
| 1CMT | COMPOSITE | 6 mth cum.AUC | 100 | 1 | 0.1272 | 4.794 |
| 1CMT | COMPOSITE | 6 mth cum.AUC | 100 | 4 | 0.808 | 4.862 |
| 1CMT | COMPOSITE | 6 mth cum.AUC | 100 | 12 | 0.7279 | 4.937 |

| | | | | | SRME% : | CV% : |
|---|---|---|---|---|---|---|
| MODEL | SAMP | LABEL | DOSE | WEEK | MODEL | MODEL |
| 1CMT+M | SERIAL | 6 mth CMAX | 10 | 1 | 12.84 | 2.748 |
| 1CMT+M | SERIAL | 6 mth CMAX | 10 | 4 | 12.57 | 2.88 |
| 1CMT+M | SERIAL | 6 mth CMAX | 10 | 12 | 13.02 | 3.121 |
| 1CMT+M | SERIAL | 6 mth CMAX | 30 | 1 | 12.78 | 3.111 |
| 1CMT+M | SERIAL | 6 mth CMAX | 30 | 4 | 13.11 | 3.019 |
| 1CMT+M | SERIAL | 6 mth CMAX | 30 | 12 | 12.6 | 3.09 |
| 1CMT+M | SERIAL | 6 mth CMAX | 100 | 1 | 12.62 | 3.252 |
| 1CMT+M | SERIAL | 6 mth CMAX | 100 | 4 | 13.14 | 2.978 |
| 1CMT+M | SERIAL | 6 mth CMAX | 100 | 12 | 13.02 | 3.437 |
| 1CMT+M | SERIAL | 6 mth cum.AUC | 10 | 1 | 3.455 | 3.015 |
| 1CMT+M | SERIAL | 6 mth cum.AUC | 10 | 4 | 3.266 | 3.138 |
| 1CMT+M | SERIAL | 6 mth cum.AUC | 10 | 12 | 3.178 | 3.18 |
| 1CMT+M | SERIAL | 6 mth cum.AUC | 30 | 1 | 3.387 | 3.581 |
| 1CMT+M | SERIAL | 6 mth cum.AUC | 30 | 4 | 3.396 | 3.254 |
| 1CMT+M | SERIAL | 6 mth cum.AUC | 30 | 12 | 2.9 | 3.18 |
| 1CMT+M | SERIAL | 6 mth cum.AUC | 100 | 1 | 3.306 | 3.238 |
| 1CMT+M | SERIAL | 6 mth cum.AUC | 100 | 4 | 3.021 | 3.041 |
| 1CMT+M | SERIAL | 6 mth cum.AUC | 100 | 12 | 2.919 | 3.32 |
| 1CMT+M | COMPO | 6 mth CMAX | 10 | 1 | 12.83 | 5.214 |
| 1CMT+M | COMPO | 6 mth CMAX | 10 | 4 | 13.02 | 5.138 |
| 1CMT+M | COMPO | 6 mth CMAX | 10 | 12 | 12.93 | 5.137 |
| 1CMT+M | COMPO | 6 mth CMAX | 30 | 1 | 12.9 | 5.171 |
| 1CMT+M | COMPO | 6 mth CMAX | 30 | 4 | 13.25 | 4.735 |
| 1CMT+M | COMPO | 6 mth CMAX | 30 | 12 | 12.7 | 5.002 |
| 1CMT+M | COMPO | 6 mth CMAX | 100 | 1 | 12.74 | 5.135 |
| 1CMT+M | COMPO | 6 mth CMAX | 100 | 4 | 12.55 | 4.757 |
| 1CMT+M | COMPO | 6 mth CMAX | 100 | 12 | 12.34 | 4.913 |
| 1CMT+M | COMPO | 6 mth cum.AUC | 10 | 1 | 3.005 | 4.78 |

| | | | | | | |
|---|---|---|---|---|---|---|
| 1CMT+M | COMPO | 6 mth cum.AUC | 10 | 4 | 3.895 | 4.873 |
| 1CMT+M | COMPO | 6 mth cum.AUC | 10 | 12 | 3.81 | 5.018 |
| 1CMT+M | COMPO | 6 mth cum.AUC | 30 | 1 | 3.572 | 4.78 |
| 1CMT+M | COMPO | 6 mth cum.AUC | 30 | 4 | 3.798 | 5.084 |
| 1CMT+M | COMPO | 6 mth cum.AUC | 30 | 12 | 3.044 | 4.721 |
| 1CMT+M | COMPO | 6 mth cum.AUC | 100 | 1 | 3.439 | 4.822 |
| 1CMT+M | COMPO | 6 mth cum.AUC | 100 | 4 | 3.656 | 5.019 |
| 1CMT+M | COMPO | 6 mth cum.AUC | 100 | 12 | 3.076 | 5.491 |

| MODEL | SAMP | LABEL | DOSE | WEEK | SRME% : MODEL | CV% : MODEL |
|---|---|---|---|---|---|---|
| 2CMT | SERIAL | 6 mth CMAX | 10 | 1 | 0.6298 | 2.803 |
| 2CMT | SERIAL | 6 mth CMAX | 10 | 4 | 1.158 | 3.014 |
| 2CMT | SERIAL | 6 mth CMAX | 10 | 12 | 0.6211 | 3.021 |
| 2CMT | SERIAL | 6 mth CMAX | 30 | 1 | 0.6298 | 2.803 |
| 2CMT | SERIAL | 6 mth CMAX | 30 | 4 | 1.158 | 3.014 |
| 2CMT | SERIAL | 6 mth CMAX | 30 | 12 | 0.6211 | 3.021 |
| 2CMT | SERIAL | 6 mth CMAX | 100 | 1 | 0.6298 | 2.803 |
| 2CMT | SERIAL | 6 mth CMAX | 100 | 4 | 1.158 | 3.014 |
| 2CMT | SERIAL | 6 mth CMAX | 100 | 12 | 0.6211 | 3.021 |
| 2CMT | SERIAL | 6 mth cum.AUC | 10 | 1 | 0.3169 | 2.891 |
| 2CMT | SERIAL | 6 mth cum.AUC | 10 | 4 | 0.811 | 3.139 |
| 2CMT | SERIAL | 6 mth cum.AUC | 10 | 12 | 0.6379 | 2.703 |
| 2CMT | SERIAL | 6 mth cum.AUC | 30 | 1 | 0.3169 | 2.891 |
| 2CMT | SERIAL | 6 mth cum.AUC | 30 | 4 | 0.811 | 3.139 |
| 2CMT | SERIAL | 6 mth cum.AUC | 30 | 12 | 0.6379 | 2.703 |
| 2CMT | SERIAL | 6 mth cum.AUC | 100 | 1 | 0.3169 | 2.891 |
| 2CMT | SERIAL | 6 mth cum.AUC | 100 | 4 | 0.811 | 3.139 |
| 2CMT | SERIAL | 6 mth cum.AUC | 100 | 12 | 0.6379 | 2.703 |
| 2CMT | COMPO | 6 mth CMAX | 10 | 1 | 0.611 | 4.481 |
| 2CMT | COMPO | 6 mth CMAX | 10 | 4 | 0.4159 | 4.665 |
| 2CMT | COMPO | 6 mth CMAX | 10 | 12 | 0.8378 | 4.972 |
| 2CMT | COMPO | 6 mth CMAX | 30 | 1 | 0.611 | 4.481 |
| 2CMT | COMPO | 6 mth CMAX | 30 | 4 | 0.4159 | 4.665 |
| 2CMT | COMPO | 6 mth CMAX | 30 | 12 | 0.8378 | 4.972 |
| 2CMT | COMPO | 6 mth CMAX | 100 | 1 | 0.611 | 4.481 |
| 2CMT | COMPO | 6 mth CMAX | 100 | 4 | 0.4159 | 4.665 |
| 2CMT | COMPO | 6 mth CMAX | 100 | 12 | 0.8378 | 4.972 |
| 2CMT | COMPO | 6 mth cum.AUC | 10 | 1 | 0.9684 | 4.76 |

| | | | | | | |
|---|---|---|---|---|---|---|
| 2CMT | COMPO | 6 mth cum.AUC | 10 | 4 | 1.059 | 4.849 |
| 2CMT | COMPO | 6 mth cum.AUC | 10 | 12 | 0.6126 | 4.281 |
| 2CMT | COMPO | 6 mth cum.AUC | 30 | 1 | 0.9684 | 4.76 |
| 2CMT | COMPO | 6 mth cum.AUC | 30 | 4 | 1.059 | 4.849 |
| 2CMT | COMPO | 6 mth cum.AUC | 30 | 12 | 0.6126 | 4.281 |
| 2CMT | COMPO | 6 mth cum.AUC | 100 | 1 | 0.9684 | 4.76 |
| 2CMT | COMPO | 6 mth cum.AUC | 100 | 4 | 1.059 | 4.849 |
| 2CMT | COMPO | 6 mth cum.AUC | 100 | 12 | 0.6126 | 4.281 |
